# As Others See Us

**Published:** 1902-05

**Authors:** 


### AS OTHERS SEE US.

The committee from the Merchants' Association of New York, who visited Texas last May, have made their report. Dr. Geo. A. Soper, the sanitary member of the committee, and with whom I had several interviews, contributes to the report an extended comment upon the sanitary status, general and local, of the State, our sanitary laws, or rather, lack of them, our Great American (the only original and genuine; get the best) quarantine department, and indulges in some merited strictures upon the same. He points out how that a bomb-proof quarantine in time of a yellow fever scare costs merchants, shippers and railroads about $5,000,000.

Speaking of the quarantine, Dr. Soper says:

"It is clear that a board of health is needed in place of the single-headed office now maintained by the State, and that its scope and authority should be commensurate with the great responsibilities which should rightfully come to it. A board, based on principles successfully carried out by such organizations as those of Massachusetts, Michigan and Connecticut, for example, with such changes and modifications as are required to adapt them to the needs of Texas, would be a strong argument to the outside world that the people were laying a strong foundation for their material and permanent advancement. By the operation of a State Board of Health the health conditions of all parts of the great territory could be accurately observed, epidemic diseases most readily controlled, valuable lessons and advice to towns and cities given as to water supplies, sewerage systems, food and drug inspection, disinfection, health ordinances, garbage disposal, etc. Not the least of the duties of the State Board of Health would be the dissemination of a broader and more accurate knowledge of the principles of preventive medicine, so that the people might receive those benefits to which they may fairly lay claim as citizens of the great commonwealth."

---

The Battle Creek Sanitarium was burned recently, the main and hospital buildings. There are, however, four quite large buildings left, properly equipped, and they will accommodate between three hundred and four hundred patients. The magnificient establishment will be speedily rebuilt, and will be completed before September 1st. It is to be five stories high and about six hundred feet long, and will, if possible, exceed the former establishment in magnificence and equipment with the latest, most up-to-date apparatus, appliances and resources for the successful treatment of diseased conditions, and for the comfort, ease and pleasure of patients. The sanitarium is known all over the world. I have always thought that it would be a perfect luxury to be sick there.

---

We publish herewith the proposed new constitution as it will be presented at Dallas for adoption.

---

H. B. Platt, of Platt's Chloride fame, dropped dead at a railroad station in New York, March 28th. The business of the firm will go on as before.

---

The Meeting of the Board of Medical Examiners for the State of Texas will be held at Waco, Texas, May 13th, ult.

